# Supplementary material for: Computational signatures of uncertainty are reflected in motor cortex excitatory neurochemistry
Source: Nat Commun. 2025 Nov 4;16:9737. doi: 10.1038/s41467-025-64702-6 (PMC12586698; doi:10.1038/s41467-025-64702-6)
Supplement: Supplementary file 1 — Supplementary Infomation [file 41467_2025_64702_MOESM1_ESM.pdf]

# SUPPLEMENTARY MATERIAL

## Computational signatures of uncertainty are reflected in motor cortex excitatory neurochemistry

Nazia Jassim[1,2], Peter Thestrup Waade[3] , Owen Parsons[2], Frederike H Petzschner[4], Catarina Rua[5,6], Christopher T Rodgers[5], Simon Baron-Cohen[2], John Suckling[2], Christoph Mathys[3], Rebecca P Lawson[1]

1. Department of Psychology, University of Cambridge, UK
2. Autism Research Centre, University of Cambridge, UK
3. Interacting Minds Centre, Aarhus University, Denmark
4. Carney Institute for Brain Science, Brown University, Providence, RI, USA
5. Wolfson Brain Imaging Centre, University of Cambridge, UK
6. Perceptive Discovery, London, UK

### MRS tissue correction

The structural T1-weighted images were pre-processed using *MATLAB R2019b* and SPM12 (www.fil.ion.ucl.ac.uk/spm/software/spm12/) using default SPM12 settings for voxel-based morphometry (VBM) and tissue segmentation [1,2]. The MP2RAGE images were first aligned to an average image in MNI space, cropped to a standard bounding box, and then segmented into tissue probability maps corresponding to grey matter (GM), white matter (WM), cerebrospinal fluid (CSF), soft tissue, bone, and air. A study-specific template was created using the DARTEL function. Images were skull stripped and warped to this template. GM and WM templates were affine transformed, warped to MNI space, and applied to each participant's tissue probability images. Tissue segmentation for each VOI was completed using the stand-alone segmentation scripts from Gannet 3.1.5 [3]. Individual MRS voxel masks were first transformed from native to MNI space and then overlaid in standard space. Metabolite levels are reported with reference to the water signal (sometimes referred to as the "absolute" concentration) [4]. We corrected the absolute metabolite concentration values for inter-individual differences in GM, WM, and CSF volumes using the alpha-correction method [5].

**Supplementary Table S1. MRS quality control metrics of final included dataset.**

| VOXEL | N | SNR Mean(SD) | LINE WIDTH *Hz* Mean(SD) | ABSOLUTE CRLB Mean(SD) | METAB-OLITE | CONCENTRATION *mmol /l* Mean(SD) | |
|---|---|---|---|---|---|---|---|
| | | | | | | Tissue-Uncorrected | Tissue-Corrected |
| **M1 (VOI)** | 37 | 79.5(15.9) | 11.7(1.1) | 0.30(0.07) | Glx | 13.4(1.08) | 14.0(1.04) |
| | | | | | GABA | 2.7(0.8) | 2.8(0.9) |
| **Occipital (Control)** | 31 | 72.5(11.4) | 13.52(0.9) | 0.30(0.05) | Glx | 13.7(1.3) | 14.1(1.2) |

VOI= Voxel of interest, SNR= Signal-Noise Ratio, CRLB = Cramér-Rao Lower Bounds

**Categorical state-transition HGF update equations**

For a detailed introduction to the generalised HGF, please refer to the original publication [5]. Continuous nodes $x_{i,j}$ are updated as continuous value parents to binary nodes $b_{i,j}$ via the following update equations:

$$\pi = \hat{\pi} + \frac{\kappa_{bin}^2}{\hat{\pi}_{bin}}$$

Equation S1

$$\mu = \hat{\mu} + \frac{\kappa_{bin}}{\pi} \delta_{bin}$$

Equation S2

where $\mu$ and $\pi$ are the mean and precision of the belief distribution respectively. $\kappa$ is the connection strength between the node and its binary child, here fixed to the default 1. $\hat{\mu}$ and $\hat{\pi}$ are the mean and the precision of the prediction respectively.

The prediction error ($\delta$) of the binary child node is calculated as:

$$\delta_{bin} = \mu_{bin} - \hat{\mu}_{bin}$$

Equation S3

The predictions for the next trial are calculated, starting at the top of the hierarchy. Continuous nodes $x_{i,j}$ are calculated with the following standard equations:

$$\hat{\mu} = \lambda \mu + \rho$$

Equation S4

$$\hat{\pi} = \frac{1}{1/\pi_{child} + \Omega}$$

Equation S5

where $\lambda$ is the autoconnection parameter controlling the degree of autoregression, here set to the default of 1 (no autoregression). $\rho$ is the total predicted drift; in this model it is fixed to 0 as there is no constant drift being learned. $\pi_{child}$ is the precision of the child; as there is no observational noise, here it is infinite (i.e., the denominator only consists of the predicted volatility $\Omega$) . As there are no additional volatility parents, $\Omega$ only consists of the $\omega$ parameter - the general expected volatility of the environment. Note that, as $\omega$ is shared between all continuous nodes $x_{i,j}$,, the volatility is expected to be the same across transition types.

The prediction for the binary node only depends on the prediction of the parent (i.e., continuous nodes $x_{i,j}$ ):

$$\hat{\mu}_{bin} = \frac{1}{1 + exp(-\kappa_{bin}\,\hat{\mu}_{parent})}$$

Equation S6

$$\hat{\pi}_{bin} = \frac{1}{\hat{\mu}_{bin} \cdot (1 - \hat{\mu}_{bin})}$$

Equation S7

Finally, the prediction of the categorical node $c_i$ is a probability distribution consisting of the normalised predictions of all the parents tracking transitions from category $i$. Note that this normalisation is necessary because of small numerical divergences due to the approximate nature of variational inference. The mean prediction of the categorical node $c_i$ is calculated as:

$$\hat{\mu}_{c_i} = \frac{\hat{\mu}_{bin\ i,j}}{\sum_j(\hat{\mu}_{bin\ i,j})} \cdots \frac{\hat{\mu}_{bin\ i,j}}{\sum_j(\hat{\mu}_{bin\ i,j})}$$

Equation S8

where $\hat{\mu}_{c_i}$ represents the categorical probability distribution, calculated using predictions from binary node $\hat{\mu}_{bin\ i,j}$, and $\sum_j(\hat{\mu}_{bin\ i,j})$ sums the unnormalized predictions $\hat{\mu}_{bin\ i,j}$ across all categorical transitions, resulting in a valid probability distribution.

**Computational model comparison**

We fit the complete dataset (n=42 participants, n=1920 trials per participant) to three models that assess trial-by-trial learning. All models were fit with four chains and 2000 samples each. The categorical state-transition HGF (Model 1) is a hierarchical Bayesian model with a Gaussian process to model learning at different levels of abstraction. The Rescorla-Wagner (RW) model (Model 2) is a simple reinforcement learning model that assesses learning through prediction errors [6]. The Experience-Weighed Attraction (EWA) model (Model 3) is a hierarchical Bayesian learning model used to study how people adjust learning strategies based on experience; it has specifically been used in studies of probabilistic reversal learning [7–9]. While Models 2 and 3 do not use the same regression-based approach as Model 1, all three comparison models estimate trial-by-trial learning signals (prediction errors or experience-weighted values) that inform response tendencies. The point-wise log-likelihood matrices from each model were extracted, and used to calculate Pareto Smoothed Importance Sampling (PSIS) approximation of the leave-one-out cross validation (LOO) metric for model comparison [10]. The PSIS-LOO was calculated using the "Stan" [12] and "loo" [13] libraries for R. The LOO criterion (LOOIC) values and differences in expected log predictive density (ELPD) are reported in Table S2 . Lower LOOIC values indicate better model fit and predictive accuracy, while lower ELPD difference values indicate worse performance. Model 1 was the best-performing model, while Model 2 and Model 3 performed worse and indicated similar predictive performance given the close ELPD difference values and standard errors.

**Supplementary Table S2: Computational model comparison metrics.**

| | MODEL | LOOIC | | MODEL COMPARISON | |
|---|---|---|---|---|---|
| | | Estimate | SE | ELPD difference | SE difference |
| **Model 1** | HGF | -37933.1 | 5006.9 | 0.0 | 0.0 |
| **Model 2** | RW | 97494.7 | 393.5 | -67713.9 | 2532.9 |
| **Model 3** | EWA | 97457.4 | 403.6 | -67695.2 | 2534.3 |

HGF = Hierarchical Gaussian Filter, RW = Rescorla Wagner, EWA = Experience-Weighted Attraction, LOOIC = Leave-one-out cross validation, ELPD = Expected log predictive density.

**Supplementary Table S3: HGF Priors.**

| HGF PARAMETER | PRIORS (MEAN, SD) |
|---|---|
| $\boldsymbol{\beta_0}\ (\boldsymbol{Intercept})$ | Normal (log(500), 1.7) |
| $\boldsymbol{\beta_1}(\mathfrak{I})$ | Normal( 0, 2) |
| $\boldsymbol{\beta_2}(\boldsymbol{U_{expected}})$ | Normal (0, 2) |
| $\boldsymbol{\beta_3}(\boldsymbol{U_{unexpected}})$ | Normal (0, 2) |
| $\boldsymbol{\beta_4}(\boldsymbol{Post-error})$ | Normal (0, 1.5) |
| $\boldsymbol{\beta_5}(\boldsymbol{Post-reversal})$ | Normal (0, 1.5) |
| σ | Truncated (Normal( 0.05, 0.5), lower = 0) |
| **ω** | Normal (-3, 1) |

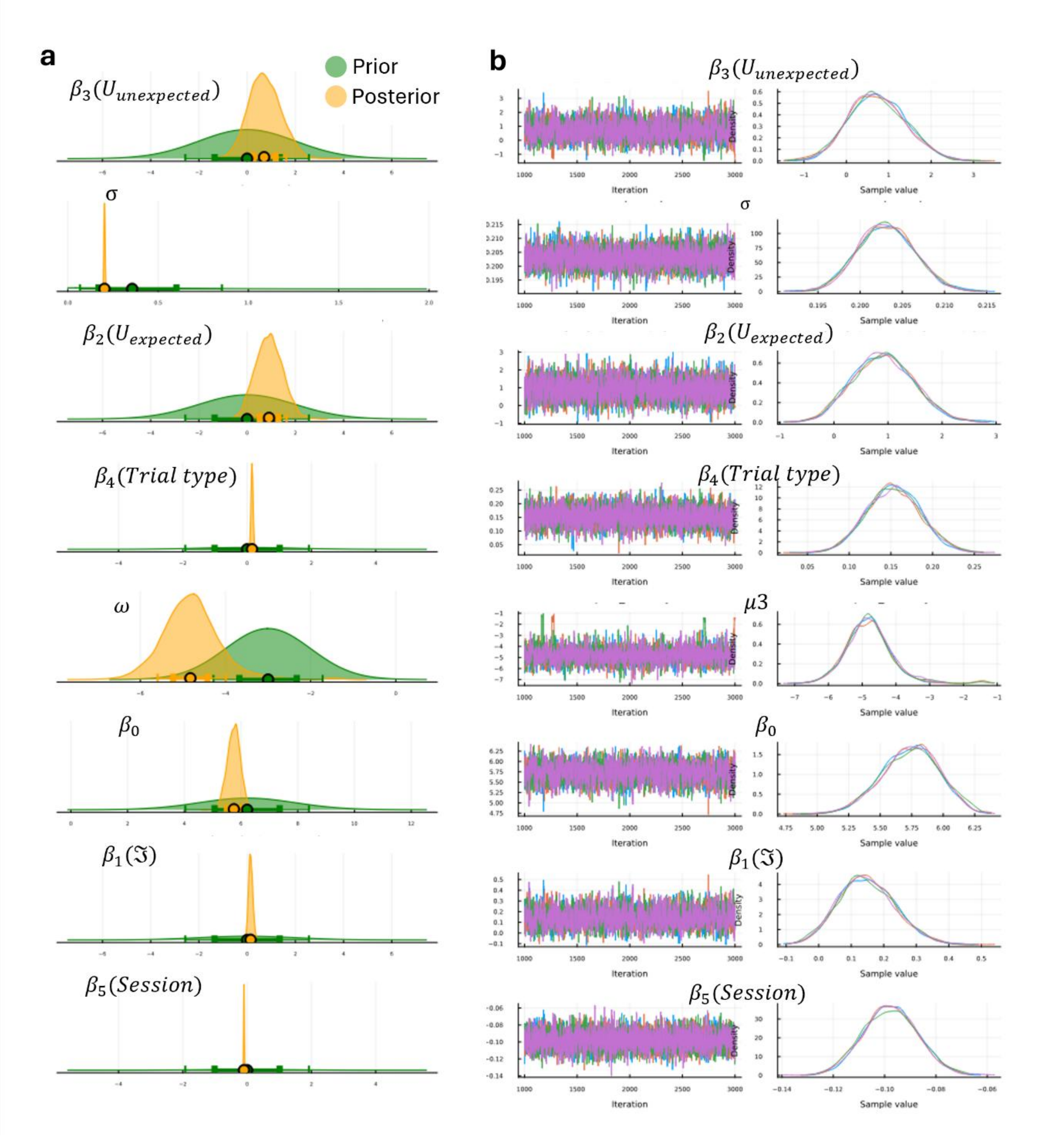


***Supplementary Figure S1. HGF model-fitting results from an example participant.*** *A) Prior (green) and posterior (orange) probability distributions for each parameter. Overlapping distributions show how the posterior is informed by the data relative to the prior. Vertical bars represent 50% and 80% credible intervals of the distribution. Circles indicate the medians. B) MCMC sampling diagnostics for the same parameters. Left subpanels show sampling chains for visual inspection of model convergence. Well-mixed and stationary traces indicate good convergence. Right subpanels show the marginal posterior densities for each chain; overlap between chains further supports convergence and reliable estimation.*

## Evolution of surprise over time

To examine how computationally-modelled surprise ($\mathfrak{I}$) evolved as a function of time, we link the model-free and model-based data through an LME model with $\mathfrak{I}$ as the dependant variable, and session (Pre- versus Post-reversal), and trial number as predictors:

$$\mathfrak{J} \sim Session * Trial\ Number + (1 + Trial\ Number |\ Participant)$$

The results revealed a significant negative slope for surprise across trials during the pre-reversal session ($t(42)=-6.54$, $p=1.01 \times 10^{-7}$, $b=-0.006$, $SE = 0.001$, 95% CI [−0.008, −0.004]), indicating that surprise decreased as participants learned the task structure. In contrast, the slope for surprise during the post-reversal session, reflected by the Session × Trial Number interaction, was not significantly different from zero ($t(42) = -0.62$, $p = 0.538$, $b = -0.001$, $SE = 0.001$, *95% CI* [−0.003,0.001]), suggesting that surprise remained relatively stable following the reversal.

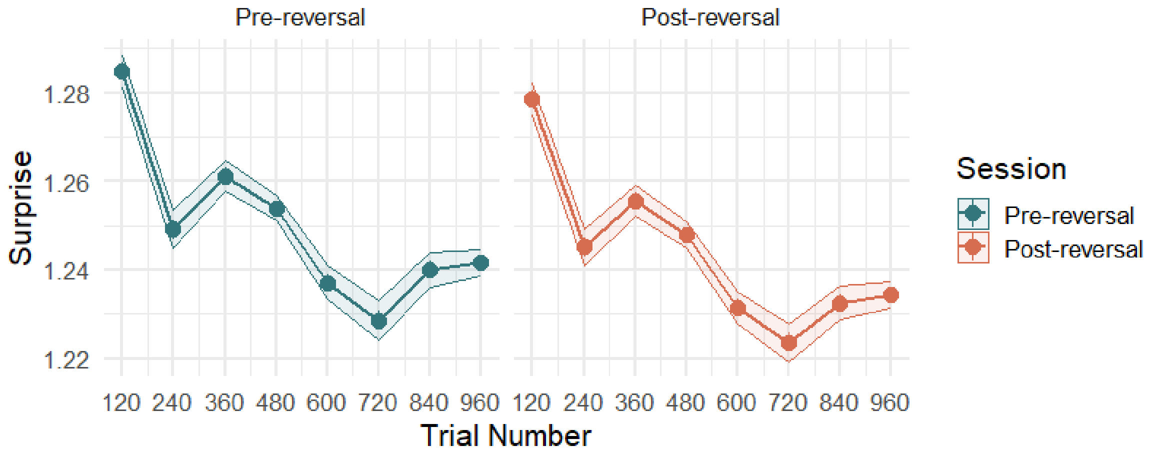


***Supplementary Figure S2. Evolution of trial-by-trial surprise ($\mathfrak{J}$) for n=42 participants, reflecting the unexpectedness of the transition relative to the participant's beliefs****. For visualization, mean $\mathfrak{J}$ (y-axis) has been calculated for 120 trial bins (x-axis), with dots displaying the means and outlines referring to the SD. The Pre-reversal session is indicated in green and the Post-reversal in orange. Source data are provided as a Source Data file.*

## Linear Mixed Effects (LME) model comparisons

For the LME models of trial-by-trial learning, we compared models including random slopes for trial number by participant to models without random slopes. This was to gauge whether there were individual differences in learning trajectories across trials. For the LME model on log RT, the inclusion of random slopes significantly improved model fit over a random intercept-only model ($\chi^2(2) = 641.36$, $p = 10^{-15}$), confirming that individuals varied meaningfully in their trial-by-trial learning patterns. Similarly, for the LME model on surprise, including random slopes also significantly improved model fit ($\chi^2(2) = 599.91$, $p = 10^{-15}$). Model fit was also evaluated using the Akaike Information Criterion (AIC) and Bayesian Information Criterion (BIC), both of which balance model fit and complexity; lower values indicate better fit, with BIC applying a stronger penalty for model complexity. The results confirm that individuals differ systematically in their trial-by-trial learning, justifying the inclusion of random slopes in the final LME models.

**Supplementary Table S4. Linear mixed-effects model comparisons.**

| LME MODEL | DEPEND-ENT VARIABLE | FIXED EFFECTS | RANDOM EFFECTS | AIC | BIC | LOG LIKE-LIHOOD | $X^2$ |
|---|---|---|---|---|---|---|---|
| **RT Model (no slopes)** | Log(RT) | (Stimulus probability + Session)* Trial Number | 1\|Participant | 40874 | 40800 | 20445 | – |
| **RT Model (with slopes)** | Log(RT) | (Stimulus Probability + Session) * Trial Number | 1 + Trial Number \| Participant | 41511 | 41418 | 20766 | 641.36 |
| **Surprise Model (no slopes)** | Surprise | Session * Trial Number | 1\|Participant | -98997 | -98941 | 49504 | – |
| **Surprise Model (with slopes)** | Surprise | Session * Trial Number | 1 + Trial Number \| Participant | -99583 | -99509 | 49800 | 590.78 |

AIC= Aikake Information Criterion, BIC = Bayesian Information Criterion

## Control MRS analyses

To rule out the possibility that the observed association between M1 Glx levels and high-level prediction errors (δ) reflects a performance-related confound, we examined the relationship between overall task accuracy and M1 Glx concentrations. This analysis revealed no significant correlation between Glx and accuracy ($r$ = 0.11, $p$ = 0.35, 95% CI [–0.13, 0.34]). These results indicate that the Glx–prediction error association is unlikely to be driven by general task performance and instead supports a more specific relationship between M1 Glx and computational learning signals.

To test for the Glx- specificity of our findings, we ran specific linear regressions with our key variables (beliefs about volatility $\omega$ and prediction errors δ) as the dependent variable and M1 metabolites (GABA and Glx) and their respective concentrations as predictors. This yielded significant interactions between M1 Glx and a) ω ($t$(62) = -2.16, $p$ = 0.03, $b$ = -0.98, $SE$ = 0.41), and b) δ (t(64) = 1.84, $p$ = 0.04, $b$ = 8.6, $SE$ = 4.68). We found no evidence of any effects of M1 GABA on the same (Fig S3 a-b). Next, to test for the regional specificity of our findings, we repeated our main correlational analyses by replacing M1 Glx with Glx concentration acquired from a control voxel. Here, we found no correlation between control Glx levels and ω ($r$ = -0.11, $p$ = 0.56, 95% CI[-0.45 0.26]), and between the control Glx levels and δ ($r$=0.14, $p$ = 0.45, 95% CI[-0.23 0.48]) (Fig S3 c-d). This confirms that Glx acquired from M1, in particular, plays a specific role in probabilistic reversal learning.

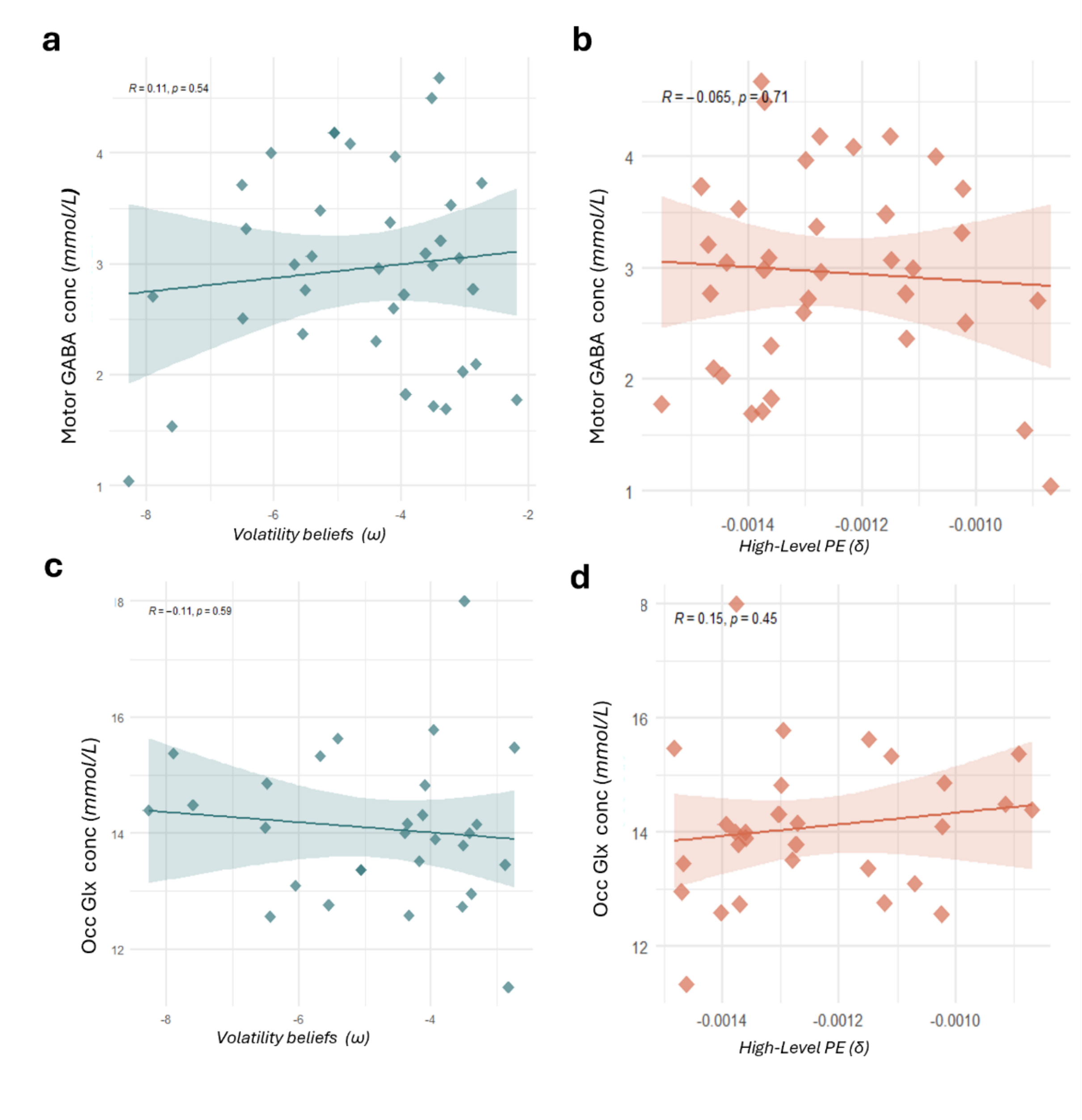


***Supplementary Figure S3. Control analyses to confirm Glx and M1 specificity of findings.*** *a) No relationship between M1 GABA and volatility beliefs (ω) (r= 0.11, p=0.54) for n=37 participants, b) No relationship between M1 GABA and high-level prediction errors (δ) (r = 0.06, p=0.71) for n=37 participants, c) No relationship between control voxel Glx and volatility beliefs(ω) (r= 0-.11, p =0.59 ) for n=29 participants. d) No relationships between control voxel Glx concentrations and high-level prediction errors (δ) (r =0.15, p=0.45) for n=29 participants. Source data are provided as a Source Data file.*

## Relationship between high-level prediction errors and volatility beliefs

A correlational analysis between participants' mean beliefs about environmental volatility and their average high-level prediction errors yielded a strong negative correlation: individuals with lower volatility beliefs exhibited larger prediction errors ($r = –0.98$, 95% CI [–0.99, –0.96], $p = 2.2 \times 10^{-16}$) (Fig S4). This finding highlights the close interplay between volatility expectations and the magnitude of prediction errors during belief updating in uncertain environments.

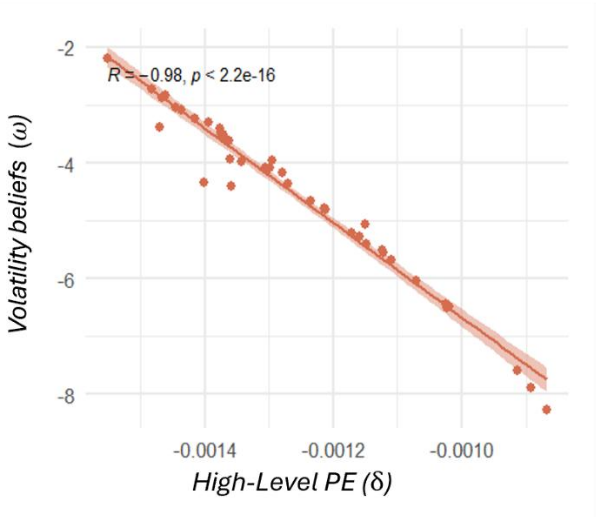


***Supplementary Figure S4.*** *Correlation between n=42 participants' mean beliefs about environmental volatility ($\omega$) and their average high-level prediction errors ($\delta$) ($r = -0.98$, , $p = 2.2 \times 10^{-16}$). Source data are provided as a Source Data file.*

## Parameter recovery

To test parameter recovery, we simulated synthetic behaviour with known generative parameters and fit the model to the parameter to see whether the generative parameters would be inferred. For each parameter of interest, for each of the input sequences of each of the 42 participants, and keeping other parameter estimates constant, behaviour was simulated over the entire range of empirically found parameter values. The medians of the posterior distributions were used as point estimates for comparing to the generative parameter values. Crucially, a strong, statistically significant correlation between the generative and recovered volatility belief ($\omega$) parameters confirmed that our key model parameter could be recovered reliably ($r = 0.92$, $p = 2.2 \times 10^{-16}$, 95% CI [0.87,0.93]) (Fig S5a). Next, we tested recovery for our key belief state surprise ($\mathfrak{I}$) by calculating $\mathfrak{I}$ values from the inputs and the parameters and using them to simulate behaviour. We found strong positive correlations between the generative and recovered values for $\mathfrak{I}$ ($r = 0.99$, $p = 2.2\text{x}10^{-16}$, 95% CI[0.99, 0.99]) (Fig S5b). Finally, we also confirmed the key response model beta-estimates could be recovered well; this was tested for post-error slowing ($r$=0.96, $p = 2.2 \times 10^{-16}$, 95% CI[ 0.96 , 0.97]), expected uncertainty ($r$= 0.99, 95% CI[0.99, 0.99], $p = 2.2 \times 10^{-16}$), and unexpected uncertainty ($r$ = 0.99, $p = 2.2 \times 10^{-16}$ , 95% CI[ 0.99, 0.99]) (Fig S3 c-e).

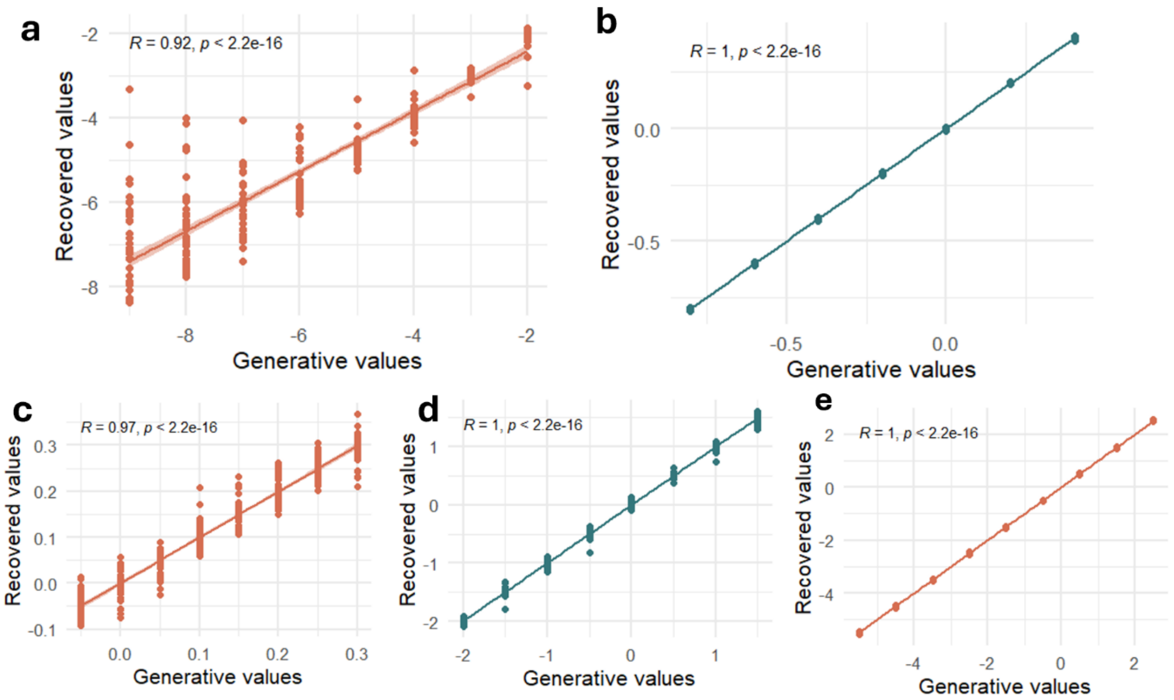


***Supplementary Figure S5.*** *Correlation between generative parameter values used to simulate n=42 datasets (generative values) and the median posteriors recovered from the model-fitting (recovered values). a) Correlation between generative and recovered values for volatility beliefs parameter (ω) (r=0.92, p=2.2x $10^{-16}$) , b) Correlation between generative and recovered values for belief state surprise (ℑ) (r=0.99, p=2.2x $10^{-16}$), c) Correlation between generative and recovered values for response model beta estimate for post-error slowing ( r=0.97, p=2.2x $10^{-16}$), d) Correlation between generative and recovered values for response model beta estimate expected uncertainty (r=0.99, p=2.2x $10^{-16}$) , e) Correlation between generative and recovered values for response model beta estimate unexpected uncertainty(r=0.99, p=2.2x $10^{-16}$). Source data are provided as a Source Data file.*

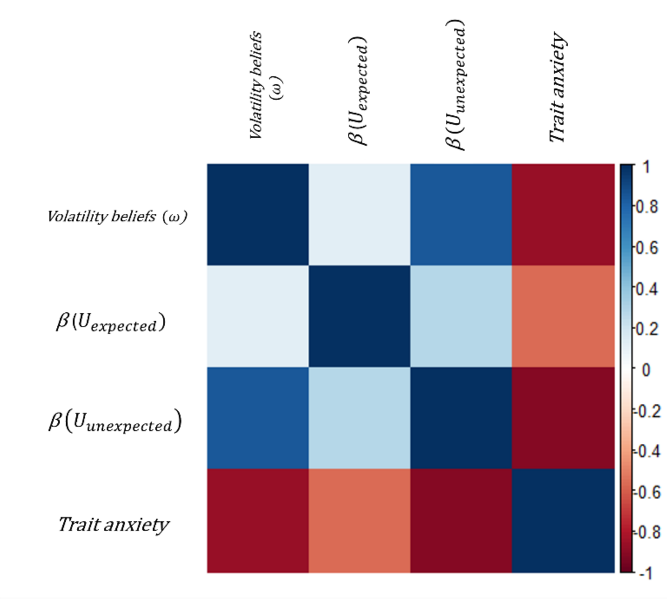


***Supplementary Figure S6.*** *Correlation matrix showing associations between trait anxiety (Spielberger State-Trait Anxiety Inventory subscale scores) and mean model-derived uncertainty measures: beliefs about volatility (ω), beta estimates for expected uncertainty ($\beta.U_{expected}$) , and unexpected uncertainty ($\beta.U_{unexpected}$) for n=42 participants.*

## Supplementary References


1. Ashburner J, Friston KJ. Voxel-Based Morphometry—The Methods. NeuroImage. 11(6), 805–21, (2000).
2. Edden RAE, Puts NAJ, Harris AD, Barker PB, Evans CJ. Gannet: A batch-processing tool for the quantitative analysis of gamma-aminobutyric acid–edited MR spectroscopy spectra. J Magn Reson Imaging. 40(6), 1445–52, (2014).
3. Lin A, Andronesi O, Bogner W, Choi IY, Coello E, Cudalbu C, et al. Minimum Reporting Standards for in vivo Magnetic Resonance Spectroscopy (MRSinMRS): Experts' consensus recommendations. NMR Biomed. 34(5), e4484, (2021).
4. Harris AD, Puts NAJ, Edden RAE. Tissue correction for GABA-edited MRS: Considerations of voxel composition, tissue segmentation, and tissue relaxations. J Magn Reson Imaging. 42(5), 1431–40, (2015).
5. Weber LA, Waade PT, Legrand N, Møller AH, Stephan KE, Mathys C. The generalized Hierarchical Gaussian Filter. Preprint at https://arxiv.org/abs/2305.10937 (2024).
6. Rescorla R, Wagner AR. A theory of Pavlovian conditioning: Variations in the effectiveness of reinforcement and nonreinforcement. In. (1972).
7. Ahn WY, Haines N, Zhang L. Revealing Neurocomputational Mechanisms of Reinforcement Learning and Decision-Making With the hBayesDM Package. Comput Psychiatry. 1(0), 24, (2017).
8. Camerer C, Hua Ho T. Experience-weighted Attraction Learning in Normal Form Games. Econometrica. 67(4), 827–74, (1999).
9. den Ouden HEM, Daw ND, Fernandez G, Elshout JA, Rijpkema M, Hoogman M, et al. Dissociable Effects of Dopamine and Serotonin on Reversal Learning. Neuron. 80(4), 1090–100, (2013).
10. McElreath R. Statistical Rethinking: A Bayesian Course with Examples in R and Stan. Chapman and Hall/CRC. (2018).
11. Stan Development Team. RStan: the R interface to Stan. R package version 2.32.6. (2024).
12. Vehtari A, Gelman A, Gabry J. Practical Bayesian model evaluation using leave-one-out cross-validation and WAIC. Stat Comput. 27(5), 1413–32, (2017).
