# Supplementary material for: Computational signatures of uncertainty are reflected in motor cortex excitatory neurochemistry
Source: Nat Commun. 2025 Nov 4;16:9737. doi: 10.1038/s41467-025-64702-6 (PMC12586698; doi:10.1038/s41467-025-64702-6)
Supplement: Supplementary file 2 — Reporting Summary [file 41467_2025_64702_MOESM2_ESM.pdf]

nature portfolio

Corresponding author(s): Nazia Jassim

Last updated by author(s): Sep 12, 2025

Reporting Summary

Nature Portfolio wishes to improve the reproducibility of the work that we publish. This form provides structure for consistency and transparency in reporting. For further information on Nature Portfolio policies, see our Editorial Policies and the Editorial Policy Checklist.

Statistics

For all statistical analyses, confirm that the following items are present in the figure legend, table legend, main text, or Methods section.

n/a	Confirmed	
☐	☒	The exact sample size (*n*) for each experimental group/condition, given as a discrete number and unit of measurement
☐	☒	A statement on whether measurements were taken from distinct samples or whether the same sample was measured repeatedly
☐	☒	The statistical test(s) used AND whether they are one- or two-sided<br>*Only common tests should be described solely by name; describe more complex techniques in the Methods section.*
☐	☒	A description of all covariates tested
☐	☒	A description of any assumptions or corrections, such as tests of normality and adjustment for multiple comparisons
☐	☒	A full description of the statistical parameters including central tendency (e.g. means) or other basic estimates (e.g. regression coefficient) AND variation (e.g. standard deviation) or associated estimates of uncertainty (e.g. confidence intervals)
☐	☒	For null hypothesis testing, the test statistic (e.g. *F*, *t*, *r*) with confidence intervals, effect sizes, degrees of freedom and *P* value noted<br>*Give P values as exact values whenever suitable.*
☐	☒	For Bayesian analysis, information on the choice of priors and Markov chain Monte Carlo settings
☒	☐	For hierarchical and complex designs, identification of the appropriate level for tests and full reporting of outcomes
☐	☒	Estimates of effect sizes (e.g. Cohen's *d*, Pearson's *r*), indicating how they were calculated

Our web collection on statistics for biologists contains articles on many of the points above.

Software and code

Policy information about availability of computer code

Data collection

The task was presented using Psychtoolbox version 3 in Matlab R2019b and created using custom code (available here: github.com/naziajassim/neurochem_markers_uncertainty). MRS data were collected using a Siemens 7T Magnetom Terra scanner (Siemens, Erlangen, Germany) with a single-channel transmit and 32-channel receiver head coil (Nova Medical, Carson, CA).

Data analysis

Behavioural data analyses and post-hoc analyses were completed in R 4.3.3 using custom scripts (github.com/naziajassim/computational_signatures_uncertainty) and R packages "lme4" and "lmerTest". The HGF models were built and fit using the novel open source Julia libraries HierarchicalGaussianFiltering.jl v0.5.4 (github.com/ComputationalPsychiatry/HierarchicalGaussianFiltering.jl) and ActionModels.jl v0.5.4 (github.com/ilabcode/ActionModels.jl). The custom scripts used for the HGF models for this particular study have been made publicly available (github.com/naziajassim/hgf_srt). The MR data were analysed using open source GUI-based software: 1) MRspa for preprocessing (www.cmrr.umn.edu/downloads/mrspa), and 2) LCModel v6.31 for quantification (http://s-provencher.com/lcmodel.shtml)

A code availability statement has been included in the manuscript. Relevant code has been shared to the following repositories:
1. Jassim, N, & Waade, PT. naziajassim/hgf_srt: Categorical state-transition HGF code for SRT task v1.1. Zenodo. Code. https://doi.org/10.5281/zenodo.17080381 (2025).
9
2. Jassim, N. naziajassim/computational_signatures_uncertainty: Comp signatures uncertainty code v1.1. Zenodo. Code. https://doi.org/10.5281/zenodo.17080373 (2025).

For manuscripts utilizing custom algorithms or software that are central to the research but not yet described in published literature, software must be made available to editors and reviewers. We strongly encourage code deposition in a community repository (e.g. GitHub). See the Nature Portfolio guidelines for submitting code & software for further information.

Data

Policy information about availability of data

All manuscripts must include a data availability statement. This statement should provide the following information, where applicable:

- Accession codes, unique identifiers, or web links for publicly available datasets
- A description of any restrictions on data availability
- For clinical datasets or third party data, please ensure that the statement adheres to our policy

The datasets analysed and modelled during the current study are publicly available:
Jassim N, Waade PT, Parsons O, Petzschner FH, Rua C, Rodgers CT, Baron-Cohen S, Suckling J, Mathys C, Lawson RP. Computational signatures of uncertainty are reflected in motor cortex excitatory neurochemistry. figshare. Dataset. https:/doi.org/10.6084/m9.figshare.28430543.v1 (2025).
Source data are provided with this paper.

A Data Availability statement has been included in the manuscript.

Research involving human participants, their data, or biological material

Policy information about studies with human participants or human data. See also policy information about sex, gender (identity/presentation), and sexual orientation and race, ethnicity and racism.

Reporting on sex and gender	Participants were asked to report both biological sex assigned at birth and gender. These details were collected primarily to ensure a sex-balanced sample. Sex/gender was not considered as a variable of interest or covariate in this study.
Reporting on race, ethnicity, or other socially relevant groupings	We did not collect information on nor do we report on race, ethnicity, or other social groups.
Population characteristics	43 right-handed participants (20 Female: 23 Male), aged 19-39 years (Mean=28.37, SD= 4.75).
Recruitment	Participants were recruited through social media, local online classifieds, University mailing lists, physical posters at local venues, and word of mouth.
Ethics oversight	This study was approved by and conducted in accordance with the regulations of the University of Cambridge Psychology Research Ethics Committee (PRE.2020.127).

Note that full information on the approval of the study protocol must also be provided in the manuscript.

Field-specific reporting

Please select the one below that is the best fit for your research. If you are not sure, read the appropriate sections before making your selection.

☐ Life sciences ☒ Behavioural & social sciences ☐ Ecological, evolutionary & environmental sciences

For a reference copy of the document with all sections, see nature.com/documents/nr-reporting-summary-flat.pdf

Behavioural & social sciences study design

All studies must disclose on these points even when the disclosure is negative.

Study description	Quantitative experimental design.
Research sample	The participant group was a random sample recruited from the general population. For convenience, they were local to or within commutable distance to Cambridge, UK. Approximately half the sample consisted of students and half were employed full-time. All participants had a minimum of high school/ secondary school level education. The final included sample consisted of 43 right-handed participants (20 Female: 23 Male), aged 19-39 years (Mean=28.37, SD= 4.75). All participants provided informed consent. Participants were paid £40 for their time.
Sampling strategy	We used a random sampling strategy. This approach ensures that the sample is representative of the targeted population of healthy adults. The sample was chosen to investigate the neural and computational mechanisms of implicit probabilistic learning and motor cortex neurochemistry, allowing for high-quality MRS data collection and adequate statistical power based on prior studies using similar paradigms. The sample size was chosen based on a power calculation (details have been reported in the manuscript).<br><br>Our study required participants to undergo MRI scanning, which may introduce self-selection bias. Individuals with claustrophobia, metal implants, or other contraindications were excluded, and participants willing to complete an MRI session may differ in anxiety, comfort with confined spaces, or general health compared to the broader population. While these factors could influence recruitment and sample composition, they are unlikely to affect the internal validity of the observed relationships between neurochemical assessments, computational variables, and behaviour.

Data collection	All data were collected in person in a laboratory setting with a researcher present at all times. There was no researcher blinding. Participants completed the task on a desktop computer at a viewing distance of 50 cm from the screen in a darkened room. The MRI scan was completed at the Wolfson Brain Imaging Centre, University of Cambridge, using a Siemens 7T Magnetom Terra scanner. In addition to the researcher, up to two radiographers were present during the scan.
Timing	All data were collected between June- September 2021. The main factors contributing to the timing of the data collection period were scheduling, scanner/testing room availability, and delays due to COVID-19 (i.e., cancellations/rescheduling due to infections).
Data exclusions	MRS data were excluded based on predetermined quality control metrics in line with the current best practices as reported in the manuscript. As a result of data exclusions, data from 37 participants remained for the MRS analyses.
Non-participation	One participant dropped out at the MRI scan stage of the study due to claustrophobia.
Randomization	There was no randomization or group allocation as the study examines brain-behaviour relationships in a single group of participants.

Reporting for specific materials, systems and methods

We require information from authors about some types of materials, experimental systems and methods used in many studies. Here, indicate whether each material, system or method listed is relevant to your study. If you are not sure if a list item applies to your research, read the appropriate section before selecting a response.

Materials & experimental systems

n/a	Involved in the study	
☒	☐	Antibodies
☒	☐	Eukaryotic cell lines
☒	☐	Palaeontology and archaeology
☒	☐	Animals and other organisms
☒	☐	Clinical data
☒	☐	Dual use research of concern
☒	☐	Plants

Methods

n/a	Involved in the study	
☒	☐	ChIP-seq
☒	☐	Flow cytometry
☐	☒	MRI-based neuroimaging

Plants

Seed stocks	n/a
Novel plant genotypes	n/a
Authentication	n/a

Magnetic resonance imaging

Experimental design

Design type	Structural MRI and MR spectroscopy (no functional MRI were acquired).
Design specifications	The following data were acquired in a single one hour MRI session while participants were at rest: structural MRI, MRS of motor cortex (voxel of interest), and MRS of occipital cortex (control voxel).
Behavioral performance measures	No behavioural data were collected during the scan. The task was completed prior to the scan. Data were collected by means of button presses. Reaction times and accuracy rates were assessed. Behavioural data outliers were identified as RT < 200 ms, while slow RT outliers were computed separately for each participant as trials with RT more than 2 standard deviations from their overall RT.

Acquisition

Imaging type(s)	Structural MRI and MR spectroscopy
Field strength	7 Tesla
Sequence & imaging parameters	T1-weighted MP2RAGE structural scans (repetition time = 4300 ms, echo time = 1.99 ms, bandwidth= 250 Hz/px, voxel size = 0.75 mm3, isotropic field of view = 240 x240x157 mm, acceleration factor = 3, flipangle = 5/6° and inversion times = 840/2370 ms) were acquired. Spectra were acquired using a short-echo semi-LASER sequence with repetition time/ echo time = 5000/26 ms, 64 repetitions. Pre-scan optimisation included FASTESTMAP, B0-shimming, unsuppressed water-peak series B1 calibration, and VAPOR water suppression calibration.
Area of acquisition	Whole brain structural MRI data were collected, while MRS data were acquired from specific voxels. MR spectra were obtained from a 2x2x2 cm3 voxels. Voxels were manually placed while participants were at rest. The voxel of interest (VOI), the primary motor cortex (M1), was centred on the omega- or epsilon-shaped left hand knob area using the central sulcus as a landmark. The occipital voxel (control) was placed on the left hemisphere using the calcarine sulcus as a landmark and positioned parallel to the cerebellum. The calcarine sulcus landmark could be most clearly seen in the sagittal slice and could be seen forming a characteristic "Y" or triangular shape with the parieto-occipital sulcus. Both voxels were positioned to include as much grey matter as possible.

Diffusion MRI ☐ Used ☒ Not used

Preprocessing

Preprocessing software	The acquired MR spectra were preprocessed using MRspa (www.cmrr.umn.edu/downloads/mrspa/). The structural T1-weighted images were pre-processed using MATLAB R2019b and SPM12 (www.fil.ion.ucl.ac.uk/spm/software/spm12/) using default SPM12 settings for voxel-based morphometry (VBM).
Normalization	The MP2RAGE images were aligned to an average image in MNI space and cropped to a standard bounding box. Individual MRS voxel masks were first transformed from native to MNI space and then overlaid in standard space.
Normalization template	A study-specific template was created using the SPM12 DARTEL function. Images were skull stripped and warped to this template. GM and WM templates were affine transformed, warped to MNI space, and applied to each participant's tissue probability images. Tissue segmentation for each VOI was completed using the stand-alone segmentation scripts from Gannet 3.1.5.
Noise and artifact removal	The MRS data were corrected for eddy current effects, and phase and frequency drifts.
Volume censoring	Default SPM12 settings for VBM were used.

Statistical modeling & inference

Model type and settings	Metabolites between 0.5 and 4.2 ppm – including Glx and GABA- were quantified using LCModel v6.31. Voxel-based morphometry (VBM) of the structural MRI was done using MATLAB R2019b and SPM12. Tissue segmentation for each VOI was completed using the stand-alone segmentation scripts from Gannet 3.1.5 3. Metabolite levels are reported w ith reference to the water signal (sometimes referred to as the "absolute" concentration). The absolute metabolite concentration values were corrected for inter-individual differences in GM, WM, and CSF volumes.
Effect(s) tested	Baseline MRS data were acquired while participants were at rest. The quantified neurochemicals were then used as variables of interest in the analyses to examine brain-behaviour relationships.

Specify type of analysis: ☐ Whole brain ☒ ROI-based ☐ Both

Anatomical location(s)	Voxels were manually placed during acquisition. See acquisition section above for details of anatomical landmarks used.
Statistic type for inference (See Eklund et al. 2016)	n/a
Correction	n/a

Models & analysis

n/a	Involved in the study	
☒	☐	Functional and/or effective connectivity
☒	☐	Graph analysis
☒	☐	Multivariate modeling or predictive analysis
